# SWOT analysis of collaborative research among family physicians in Ghana: A workshop report

**DOI:** 10.4102/phcfm.v17i1.4804

**Published:** 2025-01-21

**Authors:** Stephen T. Engmann, Nana K. Ayisi-Boateng, Dora Egblewogbe, Priscilla Vandyck-Sey, Baaba N. Damoah, George B. Nketiah

**Affiliations:** 1 Society of Family Physicians of Ghana, Accra, Ghana; 2 Family Medicine Unit, Manna Mission Hospital, Accra, Ghana; 3 Department of Medicine, Kwame Nkrumah University of Science and Technology, Kumasi, Ghana; 4 Family Medicine Unit, The Trust Hospital Company Limited, Accra, Ghana; 5 Korle Bu Polyclinic, Department of Family Medicine, Korle Bu Teaching Hospital, Accra, Ghana

**Keywords:** research, collaboration, family physicians, scientific workshop, Ghana

## Introduction

Conducting research studies and knowledge management are central to enhancing quality primary health care.^1^ The provision of accessible and patient-oriented healthcare remains a global challenge despite actions taken by family physicians to strengthen primary healthcare.^1^ Good clinical research collaboration seeks to improve client care through evidence-based practice, thus strengthening population health as a whole. Research projects involving coordination between researchers, institutions, organisations and/or communities are referred to as collaborative research.^2^ The potential benefits of collaborative research include interdisciplinary exchange of ideas, new skills acquisition, access to funding and improved research outcomes.^2^ Healthcare services continue to face a significant barrier in their ability to integrate newly obtained information and research findings into routine practice.^3^ There is therefore a need for dedicated continuous efforts towards research collaboration among various organisations to optimise health systems through clinical practice and training.^4^ At the 10th Annual General and Scientific Meeting (AGSM) of the Society of Family Physicians of Ghana (SOFPOG) in May 2024, a workshop on collaborative research was held for family physicians from across the country. This report is a summary of the workshop findings. The objective of the conference workshop was to explore strengths, weaknesses, opportunities and threats (SWOT) related to collaborative research among family physicians in Ghana and generate recommendations for enhanced collaborative research by family physicians.

## Workshop process

The workshop was organised on 17 May 2024, in the Eastern Region of Ghana as part of the events of the 10th AGSM of SOFPOG. The aim of this workshop was to identify the strengths, weaknesses, opportunities and threats to collaborative research among family physicians. The workshop began with a presentation on an overview of collaborative research. Following this, there was a breakout session, and participants were randomly placed in four groups, based on each component of the SWOT analysis; Group 1 – Strengths, Group 2 – Weaknesses, Group 3 – Opportunities and Group 4 – Threats. Each group had discussions on these headings led by a facilitator and assisted by a rapporteur. Senior faculty members from tertiary institutions and/or accredited training institutions were also assigned to all four groups. Key concepts from the four groups were summarised by the facilitators.

## Workshop findings

### Participants

The workshop had a total of 85 attendees who were all members of SOFPOG at various levels of their careers; family medicine residents, experienced family physicians some of whom are trainers, as well as early and mid-career family physicians. Participants were from most of the family medicine-accredited training centres as well as five major teaching hospitals in the country. There were conference participants who came from Komfo Anokye Teaching Hospital (KATH), Korle-Bu Teaching Hospital (KBTH), Cape Coast Teaching Hospital (CCTH), Tamale Teaching Hospital (TTH) and the Ho Teaching Hospital (HTH), all of which are family medicine post-graduate training centres. By the year 2023, there were eight family medicine post-graduate membership training centres in the country.^5^

### Strengths

Among the strengths identified was the high diversity of knowledge and backgrounds among members of SOFPOG, which enhances collaborative research efforts and allows convenient access to primary data. The wide distribution of family physicians across the nation contributes to the generation of data in various areas, enabling more decentralised and varied studies. Collaborative research among members of SOFPOG, especially when experienced researchers are involved, not only enhances the credibility of the work but also attracts financial benefits, such as access to grants. Additionally, collaborative research fosters mentorship opportunities, with more experienced members guiding younger researchers to improve their research capabilities. Family physicians are also positioned to give suggestions of relevant research areas based on current disease trends seen in practice.

Family physicians, as gatekeepers of primary care, maintain a direct influence on the well-being of the communities they care for, and hence their participation in research will guarantee primary access to data and generate findings that can have a positive impact on the community. With access to large volumes of high-quality data from primary care, which is the bedrock of healthcare delivery, family physicians are strategically positioned to collaborate among themselves and with other categories of health workers, scientists and researchers.

### Weaknesses

Among the weaknesses identified in conducting collaborative research among family physicians in Ghana was the inadequate knowledge of the research process among certain physicians from the undergraduate medical school level, which carries over into their postgraduate education. Other weaknesses identified were time limitations to do collaborative research, a lack of interest in research by some doctors, financial constraints and inadequate research exposure resulting from a lack of effective mentorship. Most family physicians in Ghana have extremely heavy clinical responsibilities and managerial roles with limited time available for collaborative research. Just like other low- and middle-income countries (LMICs), doctors in Ghana usually have limited funding for conducting research, coupled with inadequate knowledge of the sources from which grants and funds can be sought. From the findings on the weaknesses relating to collaborative research, the lack of interest in research by some doctors could be because of little or no motivation for research. Another challenge noticed was poor collaboration and communication between members of a research team especially when members were in different practice locations across the country.

### Opportunities

The opportunities for collaborative research identified from the workshop were the availability of faculty members well-distributed across various local and international institutions, providing a diverse, vibrant academic and clinical environment. Within the local context, public health facilities such as teaching hospitals, including the KATH, KBTH, CCTH, TTH and the HTH, as well as the 37 Military Hospital were identified. Academic institutions such as the University of Ghana Medical School (UGMS), Kwame Nkrumah University of Science and Technology (KNUST), University for Development Studies (UDS), University of Health and Allied Sciences (UHAS), and the University of Cape Coast (UCC) were also highlighted. Members of the Society serve in various capacities within the Ghana Health Service (GHS), Christian Health Association of Ghana (CHAG), Ministry of Health (MOH) and Civil Society Organisations (CSOs), with additional representation in private institutions such as Nyaho Medical Centre and the Trust Hospital Limited.

Opportunities for international collaboration were also possible with organisations such as SafeCare International, the World Organisation of Family Doctors (WONCA) and AfriWon Research Collaboration (ARC) programme. Opportunities for collaborations across institutions reflect a range of strategic partnerships aimed at enhancing healthcare delivery and research. Members of SOFPOG in teaching hospitals could partner with academic institutions, as well as other teaching hospitals to facilitate shared learning and clinical advancement.

### Threats

Collaborative research in family medicine faces several challenges, primarily because of poor recognition of family physicians’ roles by other organisations, both in academia and clinical practice. Many stakeholders are either unaware of or disinterested in family medicine, limiting their engagement in research collaborations. Furthermore, there is often insufficient support and funding for family medicine research. The demanding clinical roles of family physicians, particularly in resource-limited settings, further hinder their ability to participate in research. Competing interests from other health sectors and bureaucratic obstacles also delay research progress. There may be a high level of apathy or inertia in some institutions or among some stakeholders when it comes to supporting research.

### Recommendations

The recommendations offered by participants were categorised into two levels: organisational and individual (Box 1). At the organisational level, recommendations included highlighting the value of family medicine research to some institutions and high-ranking individuals to encourage research collaboration with family physicians and ensuring the processes for approvals of documents for research collaboration and implementation are enhanced. The Government of Ghana must be made aware of the major role family physicians play in community health, community medicine and development, and encouraged to offer adequate support for research by family physicians. There could be collaboration between SOFPOG and the faculty of family medicine of postgraduate colleges to map or develop a directory of where family physicians are located in the country to encourage working together on research projects.

BOX 1 Workshop-generated recommendations.

**Individual level**

- Involvement of residents in collaborative research right from the start of training
- More mentors to help build researchers and strengthen mentor–mentee relationships
- Time allocation by family physicians for participation in research
- Collaboration of family physicians in academic institutions with grant support and other family physicians in clinical services.
- Encourage career progression and mentorship in family medicine research and collaboration

**Organisational level**

- The government should recognise the role of family medicine in community health and support research initiatives.
- Collaboration between SOFPOG and the faculty of family medicine of postgraduate colleges to map or develop a directory to locate where family physicians are located in the country to encourage working together
- Research seminars for private and public facilities to encourage collaborations in research
- A problem list by SOFPOG to identify research gaps and general challenges in the health system that could be prioritised for research
- Build capacity for research and publications through faculty-led workshops in academic institutions

*Source:* Celebrating a Decade of Dedication: World Family Doctor Celebrations and AGSM 2024 by the Society of Family Physicians of Ghana. Recommendations presented at the 10th Annual General and Scientific Meeting (AGSM) of SOFPOG; 2024 May 16–19; Koforidua, Eastern Region, Ghana. Wonca; 2024.^6^

SOFPOG, Society of Family Physicians of Ghana.

Adequate research training should begin early in medical school and continue through membership training to the fellowship level. Young doctors should be encouraged to write case reports and supported to build their capacity in research. Family physicians should adopt good time management principles that allow some time to engage in research. Lastly, mentorship by senior family physicians should also be enhanced.

## Conclusion

The SWOT analysis revealed that family physicians in Ghana possess diverse knowledge and strong community ties, but face challenges such as inadequate research training and heavy clinical workloads. Opportunities for research collaboration exist through partnerships with local and international institutions. This report has underscored the need for enhanced engagement and support in research initiatives to improve primary healthcare delivery. To enhance collaborative research, recommendations are made for improving research training from medical school through to fellowship training, fostering mentorship relationships and establishing a directory of family physicians across the country that could facilitate collaboration on research projects. Ultimately, these efforts seek to leverage the unique position of family physicians in the healthcare system to generate impactful research that translates into better health outcomes for patients and the population.
